# Case Report: The ectopic pancreas in the adrenal glands: It was found due to elevated blood pressure and initially diagnosed as adrenal adenoma

**DOI:** 10.3389/fsurg.2022.1040711

**Published:** 2022-11-03

**Authors:** Tao Zhang, Si-fan Yin, Qi-wu Wang, Wen-bo Feng, Chang-xing Ke

**Affiliations:** ^1^ Department of Urology, The Second Affiliated Hospital of Kunming Medical University, Kunming, China; ^2^ Department of Urology, The General Hospital of Western Theater Command, Chengdu, China

**Keywords:** ectopic pancreas, adrenal glands, diagnosis, treatment, clinical features

## Abstract

**Background:**

The ectopic pancreas is a kind of congenital malformation formed during embryonic development, which has no anatomical relationship with the normal pancreas and is a rare solid disease. The ectopic pancreas in the adrenal glands is extremely rare.

**Case summary:**

A 32-year-old man was admitted to the hospital after experiencing elevated blood pressure for 2 years as well as dizziness and blurred vision for 2 weeks. He had an elevated blood pressure of 170/110 mmHg (1 mmHg = 0.133 kPa) on physical examination 2 years ago, without palpitations, chest pain, and chest tightness. Two weeks ago, he presented with dizziness and blurred vision. Blood renin and aldosterone levels were elevated. Plain CT and contrast-enhanced CT scan showed nodular thickening of the left adrenal and homogeneous enhancement, which was initially considered adrenal adenoma. The postoperative pathology supported the ectopic pancreas in the left adrenal. After 78 months of postoperative follow-up, no recurrence was observed, but his blood pressure remained persistently high.

**Conclusion:**

The ectopic pancreas occurring in the adrenal glands is extremely rare, has no specific clinical symptoms, and is mainly found for other reasons. It can easily be misdiagnosed as an adrenal adenoma. The final confirmation of the diagnosis still depends on the pathological biopsy. A great deal of reporting is still required for whether there is a correlation with elevated blood pressure.

## Introduction

The ectopic pancreas is pancreatic tissue that lacks anatomical and vascular connections to the original pancreas (1). However, it has the histological features of pancreatic acinar formation, duct development, and islets (2, 3). It is rare with the main sites located in the stomach, duodenum, and jejunum (4). It can occur at any age but is most common in those between the ages of 40 and 50 years, and more common in men than in women. The ectopic pancreas occurring in the adrenal glands is extremely rare. In this article, we retrospectively analyzed the clinical data of a patient with an ectopic pancreas in the left adrenal gland, which was preoperatively misdiagnosed with adrenal adenoma. Therefore, we analyzed the clinical features of this disease in order to further improve the understanding of the disease and thus improve the diagnosis and treatment of this disease.

## Case introduction

A 32-year-old man was admitted to the hospital after experiencing elevated blood pressure for 2 years as well as dizziness and blurred vision for 2 weeks. He had an elevated blood pressure of 170/110 mmHg (1 mmHg = 0.133 kPa) on physical examination 2 years ago, without palpitations, chest pain, and chest tightness. Two weeks ago, he presented with dizziness and blurred vision, so he went to a local hospital where his blood pressure was measured as high as 143/94 mmHg. He was given oral irbesartan 150 mg once daily. The blood pressure was controlled, but the symptoms of dizziness and blurred vision were not relieved, so he was referred to our hospital.

**Figure 1:**
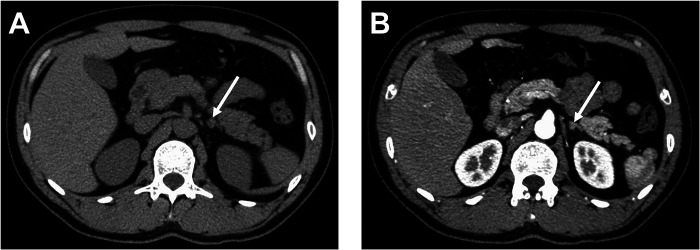
CT of patient before operation. (**A**) Plain CT. (**B**) Contrast-enhanced CT scan.

He had no specific history and denied any personal and family history associated with cancer. Physical examination revealed that no palpable masses and percussion pain were found in bilateral renal areas. The levels of serum potassium were normal (4.45 mmol/L, normal reference values: 3.5–5.3 mmol/L). Plasma normetanephrine levels were normal. The results of the clonidine test were normal (15.76 pg/ml, normal reference values: 0–100 pg/ml). Preoperative plasma renin and aldosterone levels were elevated in the supine (55.57 pg/ml and 550.98 pg/ml; reference ranges 4–24 and 10–160, respectively) and upright positions (59.96 and 667.30 pg/ml; reference ranges 4–38 and 40–310). Tests on the levels of catecholamines in the blood and urine were negative. On CT, nodular thickening of the left adrenal was detected on a plain CT scan (Figure 1A, arrow), measuring approximately 0.6 cm in the right-left diameter × 0.7 cm in anterior–posterior diameter × 0.9 cm maximal superior–inferior diameter. The CT value was approximately 45 HU, and homogeneous enhancement on the contrast-enhanced CT scan with a CT value of approximately 97 HU (Figure 1B, arrow). According to laboratory and imaging examinations, a left adrenal adenoma was considered. We changed to valsartan plus trimetazidine hydrochloride to control blood pressure.

**Figure 2:**
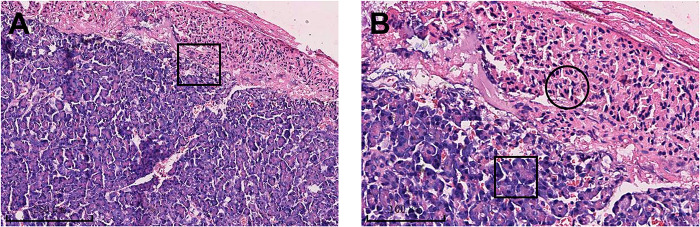
The ectopic pancreas in the left adrenal gland. There was a histological transition zone between the ectopic pancreas and the adrenal gland (**A**). Acini of ectopic pancreas (black rectangle in **B**). The adrenal gland (black circle in **B**).

Due to unsatisfactory long-term blood pressure control and a strong desire for surgery, after completing preoperative preparation, a posterior laparoscopic left adrenalectomy was performed for the left adrenal mass. Postoperative pathology supported the ectopic pancreas in the left adrenal. In histopathological examination, the mass consisted of pancreatic follicles and ducts; the ducts were irregularly dilated, hyperplastic, and lined with mucous epithelium or columnar epithelium (Figure 2). The patient made a successful recovery after the surgery. After 78 months of postoperative follow-up, no recurrence was observed, but his blood pressure remained persistently high.

Timeline: Elevated blood pressure was found for 2 years, dizziness and blurred vision for 2 weeks—Relevant laboratory tests and examinations were completed and the clinical consideration was left adrenal adenoma in the 6 hospital days—Laparoscopic resection of left adrenal occupancy was performed in the 7 hospital days—The definite histological examination confirming an ectopic pancreas in the adrenal glands was made in the 13 hospital days—The blood pressure remained persistently high in the next 78 months of postoperative follow-up.

## Related literature learning

The ectopic pancreas, also known as pancreatic heterotopia or aberrant pancreas, is defined as pancreatic tissue that lacks anatomical and vascular connections to the original pancreas. However, it has the histological features of pancreatic acinar formation, duct development, and islets (2, 3). The adrenal gland is an extremely rare location for the ectopic pancreas. Only two cases were found in the adrenal glands, including ours (5). It can occur at any age but is most common in those between the ages of 40 and 50 years, and more common in men than in women. A related study noted that the percentage of pathological findings is 0.2% in abdominal surgery, 0.9% in gastrectomies, and 0.55%–13.7% in autopsy (6). The etiology of the ectopic pancreas is unknown. There are several theories regarding the development of the ectopic pancreas, including the dislocation theory, the metaplasia theory, and the totipotent cell theory. The most commonly accepted explanation is the dislocation theory, which states that during the rotation of the foregut, the original pancreatic components are separated, deposited, and develop ectopically, resulting in the gradual formation of mature pancreatic tissue (2, 7).

The clinical presentation is nonspecific or is found due to other diseases. When accompanied by inflammation, bleeding, obstruction, or cancer, it can lead to serious clinical manifestations and complications (6). Although there are various imaging diagnostic methods such as CT and ultrasound, the imaging features of the ectopic pancreas are unclear and difficult to diagnose (8). In this case, the patient was admitted with elevated blood pressure, dizziness, and blurred vision. Plain CT and contrast-enhanced CT scan showed nodular thickening of the left adrenal and homogeneous enhancement. The majority of ectopic pancreatic attenuation is similar to or higher than that of the pancreas, probably due to its histological similarity to normal pancreatic tissue, especially the acini (8). The levels of serum potassium were normal, and the preoperative renin and aldosterone levels were elevated in the supine and upright positions. Combining the imaging and clinical manifestations, it was initially considered to be left adrenal adenoma, while postoperative pathological examination diagnosed an ectopic pancreas in the left adrenal glands.

Since lacking specific clinical manifestation or effective examination, confirmation of the diagnosis relies on postoperative pathological examination. Microscopically, the ectopic pancreatic tissue resembles the orthotopic pancreas and appears as lobular structures. Pancreatic follicles, pancreatic ducts, and even pancreatic islets are visible (9). According to the von Heinrich classification system, the ectopic pancreas can be classified into three types based on microscopy. Type 1: ectopic tissue consists of all components of normal pancreatic tissue, including acini, ducts and islets; Type 2: ectopic tissue consists of acini and ducts without islets; Type 3: ectopic tissue consists of ducts only (10, 11). In this case, the pancreatic acini and ducts were visible microscopically, and there was no islet, which was a type II ectopic pancreas.

The preferred surgical treatment, especially with the development of minimally invasive techniques. Laparoscopic resection has become the gold standard for adrenal occupancy. However, there is still some controversy about the need for surgical treatment in people with no obvious symptoms. Most scholars believe that the ectopic pancreas has potential pathogenic and even malignant risks, and once diagnosed, surgery should be performed regardless of whether there are symptoms or not (3). If an ectopic pancreas is incidentally found intraoperatively, it is advisable to remove it at the same time as far as possible without interfering with the scheduled surgery (11). This case was underwent laparoscopic resection. During the postoperative follow-up, the patient's blood pressure remained high. He was followed up at the local hospital, while we asked the patient by telephone without aldosterone and renin testing, so the cause of his elevated blood pressure still deserves to be explored.

In conclusion, the ectopic pancreas is rare and is currently reported to be located mostly in the stomach, duodenum, and jejunum. It is extremely rare to occur in the adrenal glands and has no specific clinical symptoms, but is mainly found for other reasons, and its diagnosis still depends on pathological biopsy. Our study aims to provide a rationale that the pancreas can be ectopic to the adrenal glands. When the ectopic pancreas is located in the adrenal glands, it is easily diagnosed as adrenal adenoma. A great deal of reporting is still required to determine whether there is a correlation with elevated blood pressure.

## Data Availability

The original contributions presented in the study are included in the article/Supplementary Material, further inquiries can be directed to the corresponding author.
